# Proteomic Characterization of Human Peripheral Blood Mononuclear Cells Exposed to a 50 Hz Magnetic Field

**DOI:** 10.3390/ijms26136035

**Published:** 2025-06-24

**Authors:** Massimo Bracci, Raffaella Lazzarini, Francesco Piva, Matteo Giulietti, Elena Marinelli Busilacchi, Elisa Rossi, Fabio Di Criscio, Lory Santarelli, Antonella Poloni

**Affiliations:** 1 Department of Clinical and Molecular Sciences, Polytechnic University of Marche, 60126 Ancona, Italy; e.busilacchi@staff.univpm.it (E.M.B.); e.rossi@univpm.it (E.R.); f.dicriscio@pm.univpm.it (F.D.C.); l.santarelli@staff.univpm.it (L.S.); a.poloni@staff.univpm.it (A.P.); 2 Department of Specialistic Clinical and Odontostomatological Sciences, Polytechnic University of Marche, Monte d’Ago, 60131 Ancona, Italy; f.piva@staff.univpm.it (F.P.); m.giulietti@staff.univpm.it (M.G.); 3 Hematology Clinic, Azienda Ospedaliero Universitaria delle Marche, 60126 Ancona, Italy

**Keywords:** ELF-MF, PBMCs, proteomic profile, mitochondrial ADP/ATP transport, metabolic pathways, immune function

## Abstract

Exposure to extremely low-frequency magnetic fields (ELF-MF) can induce biological alterations in human cells, including peripheral blood mononuclear cells (PBMCs). However, the molecular mechanisms and key regulatory factors underlying this cellular response remain largely unknown. In this study, we analyzed the proteomic profiles of PBMCs isolated from three human subjects. PBMCs were exposed to 50 Hz, 1 mT of ELF-MF for 24 h and compared to unexposed PBMCs from the same individuals. ELF-MF exposure altered the expression levels of several PBMC proteins without affecting cell proliferation, cell viability, or cell cycle progression. A total of 51 proteins were upregulated, 36 of which were intercorrelated and associated with the *Cellular Metabolic Process* (GO:0044237) and *Metabolic Process* (GO:0008152). Among them, solute carrier family 25 member 4 (SLC25A4), which catalyzes the exchange of cytoplasmic ADP for mitochondrial ATP across the inner mitochondrial membrane, was consistently upregulated in all ELF-MF–exposed samples. Additionally, 67 proteins were downregulated, many of which are linked to *T cell costimulation* (GO:0031295), *Cell activation* (GO:0001775), and *Immune system processes* (GO:0002376) included ASPSCR1, PCYT1A, PCYT2, QRAS, and REPS1. In conclusion, ELF-MF exposure induces metabolic reprogramming in human PBMCs, characterized by the upregulation of mitochondrial proteins and downregulation of immune-activation-related proteins, without compromising cell viability or proliferation.

## 1. Introduction

Extremely low-frequency magnetic fields (ELF-MF) can directly interact with human cells, including peripheral blood mononuclear cells (PBMCs) [1]. PBMCs—which include T and B lymphocytes, natural killer (NK) cells, monocytes, and dendritic cells—play a crucial role in the immune response. The extremely low-frequency, non-ionizing portion of the electromagnetic spectrum may alter biological functions in human cells and directly or indirectly modify the protein expression profiles of exposed cells [2,3]. Exposure to 50/60 Hz ELF-MF has been associated with various biological effects, including dysregulation of reactive oxygen species (ROS), apoptosis, mitochondrial damage, and both direct and indirect DNA damage or genotoxic effects [4,5]. ELF-MF has been shown to interact with the cell membrane, receptors, and mitochondria, inducing alterations in cellular processes such as proliferation and cell cycle progression. These cell responses to ELF-MF could be related to ROS overproduction [6]. In this context, ELF-MF acts as a cellular stressor, the direct molecular targets and mechanisms activated following exposure remain largely unknown [7,8,9]. Previously, our research group investigated the effects of 50 Hz, 1 mT ELF-MF exposure on human mammary cells, identifying the key molecular factors involved in the response, particularly those related to focal adhesion, mitochondrial function, and cellular reprogramming pathways [2,10]. Wolf and colleagues reported that human fibroblasts exposed to 50 Hz, 1 mT of ELF-MF for 72 h exhibited increased cell proliferation, altered cell cycle progression, and signs of DNA damage [11]. ELF-MF has also been shown to trigger ROS production in human PBMCs [1,6]. The aim of this study was to analyze the proteomic profile of human PBMCs exposed to 50 Hz, 1 mT ELF-MF, in order to identify the molecular processes altered by ELF-MF exposure.

## 2. Results

### 2.1. ELF-MF Does Not Affect Human PBMC Proliferation, Cell Death, or Cell Cycle Progression

**Figure 1:**
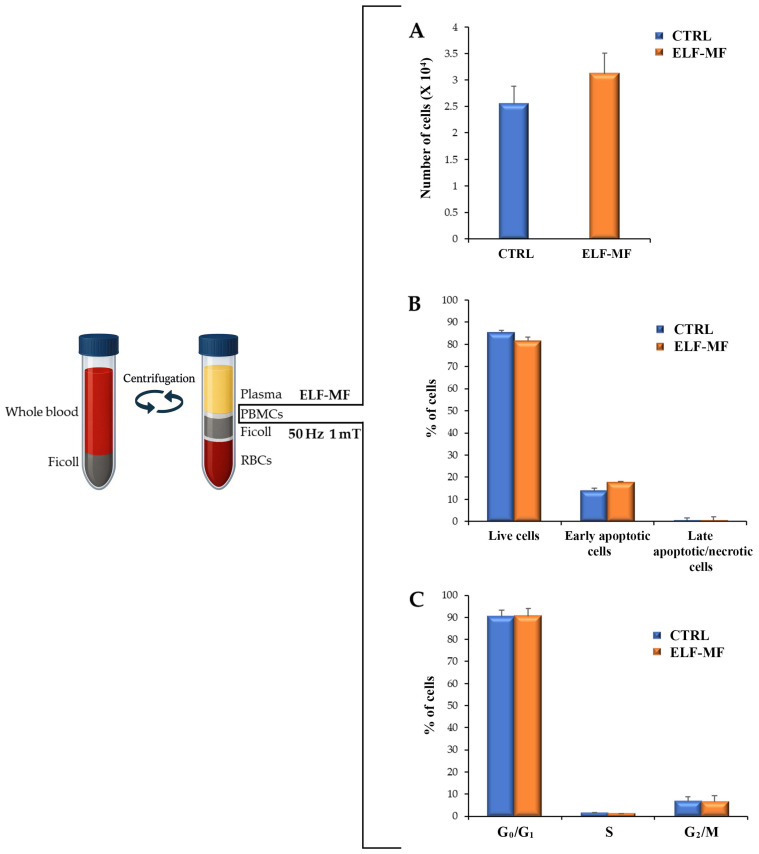
Cell number, viability, and cell cycle analysis in ELF-MF-exposed PBMCs. (**A**) The histogram shows the number of PBMCs after ELF-MF exposure, compared to unexposed samples. Cells were counted using a Bürker chamber. (**B**) Cell viability and apoptotic/necrotic rates in PBMCs exposed to ELF-MF were assessed using an Annexin V and Propidium Iodide (PI) assay, with unexposed PBMCs serving as the control. (**C**) Cell cycle analysis was performed using PI solution. Unexposed cells were used as a control. Results are presented as mean ± SD from independent experiments with individual samples.

PBMCs exposed or unexposed to ELF-MF (50 Hz, 1 mT; 24 h on/48 h off) showed no significant alterations in proliferation, viability, or apoptotic rate (Figure 1A,B). Furthermore, ELF-MF exposure did not induce necrosis in PBMCs (Figure 1B). The data also suggest that ELF-MF exposure had no significant effect on cell cycle progression in PBMCs (Figure 1C).

### 2.2. Alteration of the Proteomic Profile in PBMCs Exposed to ELF-MF

**Figure 2:**
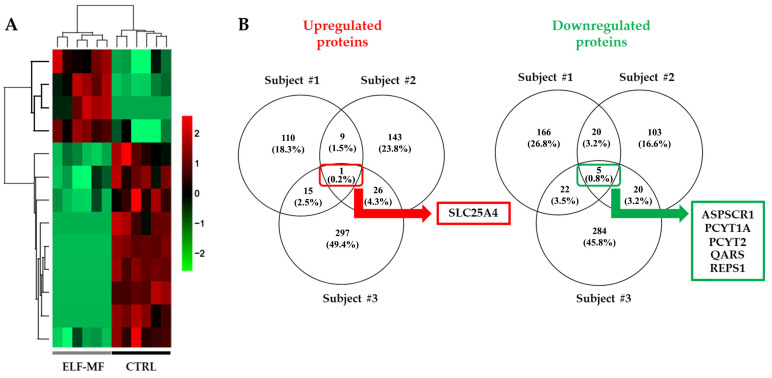
Proteomic analysis of PBMCs exposed to ELF-MF. (**A**) Hierarchical heatmap clustering showed that ELF-MF exposure altered the proteomic profile of PBMCs. Red indicates upregulation; green indicates downregulation. (**B**) The Venn diagram illustrates that ELF-MF exposure induced the upregulation of 135, 179, and 339 proteins in the PBMC samples from subject #1, subject #2, and subject #3, respectively. SLC25A4 was upregulated in all exposed samples from all three subjects. Additionally, ELF-MF exposure downregulated 213, 148, and 331 proteins in the PBMC samples from subject #1, subject #2, and subject #3, respectively. ASPSCR1, PCYT1A, PCYT2, QARS1, and REPS1 were downregulated in all exposed samples.

**Figure 3:**
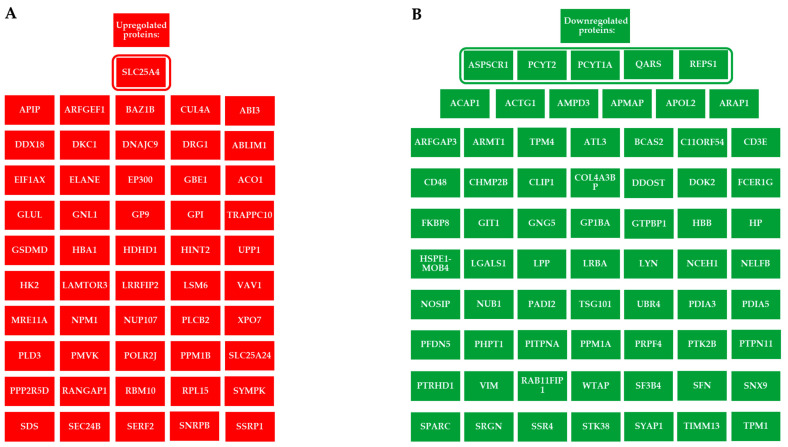
Upregulated and downregulated proteins in PBMC samples from at least two of the three subjects. (**A**) Proteomic data analysis identified 51 upregulated proteins in exposed PBMCs compared with unexposed samples. (**B**) Regarding the downregulated proteins, the analysis revealed that 67 proteins were downregulated in PBMC samples from two of the three subjects. The circled proteins were significantly upregulated or downregulated in all exposed samples.

A hierarchical clustering analysis of protein expression levels clearly revealed that ELF-MF exposure induced significant changes in the proteomic profiles of PBMCs compared with unexposed samples (Figure 2A). The Venn diagram in Figure 2B shows that ELF-MF exposure led to the upregulation of 135, 179, and 339 proteins in samples from subjects #1, #2, and #3, respectively. Notably, SLC25A4 [solute carrier family 25 (mitochondrial carrier; adenine nucleotide translocator) member 4] was upregulated in all three exposed samples (Figure 2B and Figure 3A). Additionally, 213, 148, and 331 proteins were downregulated in the exposed samples of subjects #1, #2, and #3, respectively. Five proteins were consistently downregulated across all three subjects: ASPSCR1 (tether containing UBX domain for GLUT4), PCYT1A (choline-phosphate cytidylyltransferase A), PCYT2 (ethanolamine-phosphate cytidylyltransferase), QARS1 (glutamine–tRNA ligase), and REPS1 (RalBP1-associated Eps domain-containing protein 1) (Figure 2B and Figure 3B). We also performed a differential expression analysis between the control and ELF-MF-exposed groups using the LFQ-Analyst tool (Supplementary Figures S1 and S2) [12]. The results obtained were fully consistent with the previous findings and were further refined using a Venn diagram. In particular, the volcano plot highlights the same proteins as significantly up- or downregulated across all three control samples compared to the exposed ones (Supplementary Figure S3). Additional details on expression levels are shown in Supplementary Figure S4, while fold changes and *p*-values are reported in Supplementary Table S1.

### 2.3. Cell Signaling Pathways Involved in the PBMC Response to ELF-MF Exposure

**Figure 4:**
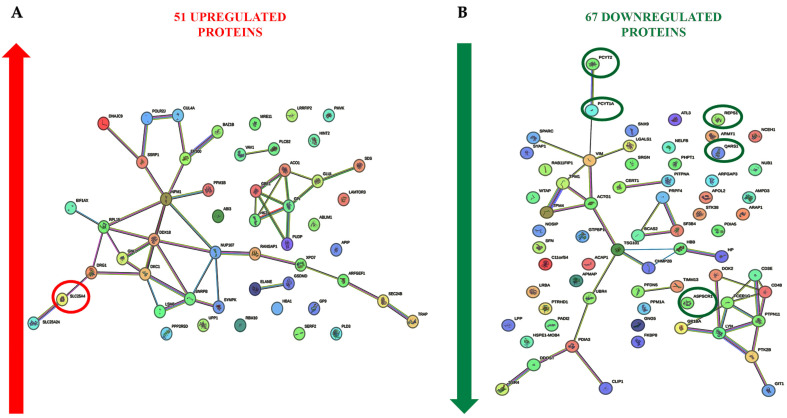
STRING analysis revealed functional associations between the 51 upregulated (red arrow) (**A**) and the 67 downregulated (green arrow) (**B**) proteins following ELF-MF exposure in PBMCs. The circled proteins were significantly upregulated (red circle) or downregulated (green circles) in all exposed samples.

**Figure 5:**
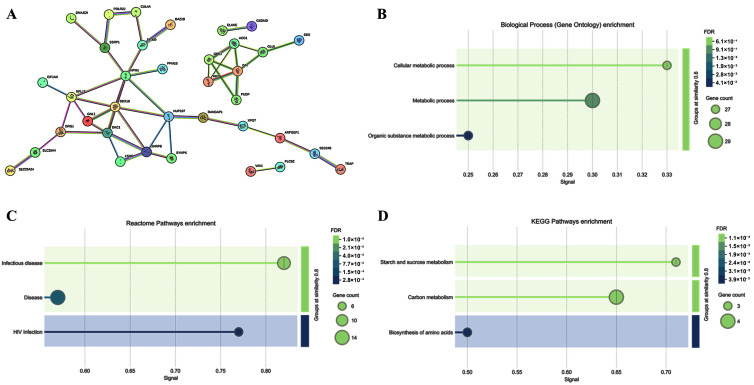
STRING analysis of 36 functionally related upregulated proteins (**A**). These proteins are involved in biological processes (Gene Ontology) such as *Cellular Metabolic Process* (GO: 0044237), *Metabolic Process* (GO: 0008152), and *Organic Substance Metabolic Process* (GO: 0071704) (**B**). They are also enriched in Reactome pathways related to *Infectious Disease*, *Disease*, and *HIV Infection* (**C**), as well as in KEGG pathways including *Starch and Sucrose Metabolism*, *Carbon Metabolism*, and the *Biosynthesis of Amino Acids* (**D**).

**Figure 6:**
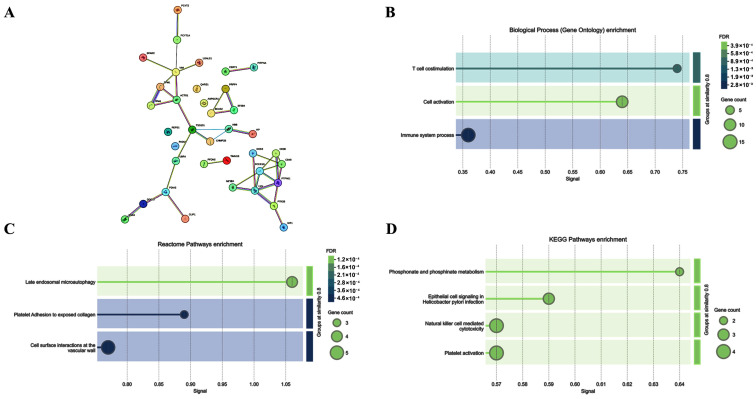
STRING analysis of 35 downregulated proteins, 32 of which are functionally related, including PCYT1A and PCYT2. The analysis also included ASPSCR1, QARS1, and REPS1, which were downregulated in all exposed samples (**A**). Biological processes (Gene Ontology) involving these proteins include *T Cell Costimulation* (GO:0031295), *Cell Activation* (GO:0001775), and *Immune System Process* (GO:0002376) (**B**). Reactome pathway enrichment analysis using the 35 downregulated proteins identified pathways such as *Late Endosomal Microautophagy*, *Platelet Adhesion to Exposed Collagen*, and *Cell Surface Interactions at the Vascular Wall* (**C**). KEGG pathway enrichment analysis revealed enrichment in *Phosphonate and Phosphinate Metabolism*, *Epithelial Cell Signaling in Helicobacter Pylori Infection*, *Natural Killer Cell-Mediated Cytotoxicity*, and *Platelet Activation* (**D**).

To increase the sensitivity of the analysis in revealing proteins potentially involved in the response to ELF-EM, we also considered those proteins up- or down-regulated in at least two of the three subjects (Figure 3A,B). ELF-MF exposure modulated the expression of several PBMC proteins, some of which were functionally interconnected, while others were not (Figure 4A,B). A bioinformatic analysis was performed on 36 upregulated, functionally related proteins (Figure 5). These included SLC25A4, which is involved in several Gene Ontology Biological Processes such as *Cellular Metabolic Process* (GO:0044237) and *Metabolic Process* (GO:0008152). SLC25A4 also participates in various Reactome pathways, including *Infectious Disease* (HSA-5663205), *HIV Infection* (HSA-162906), and *Disease* (HSA-1643685). Other upregulated proteins were associated with KEGG pathways such as *Organic Substance Metabolic Process* (GO:0071704), *Starch and Sucrose Metabolism* (hsa00500), *Carbon Metabolism* (hsa01200), and *Biosynthesis of Amino Acids* (hsa01230). Of the 67 downregulated proteins, 32 were functionally interconnected, including PCYT1A and PCYT2, which were consistently downregulated in all three PBMC samples. Additionally, ASPSCR1, QARS1, and REPS1, although not functionally clustered with others, were also downregulated in all exposed samples. Together, these 35 downregulated proteins were involved in several biological processes, including *T Cell Costimulation* (GO:0031295) and *Cell Activation* (GO:0001775) (Figure 6).

## 3. Discussion

This study investigated the effects of ELF-MF exposure on PBMCs, focusing on alterations in their proteomic profile. PBMCs were used as a model system commonly employed to study immune responses, including the acquisition of a pro-inflammatory phenotype and cytokine production in response to various stimuli [13]. These immune cells are capable of responding to external factors, including ELF-MF. In vitro studies have reported that ELF-MF can interact directly with human cells, leading to increased levels of ROS, alterations in mitochondrial morphology, and changes in the expression of genes involved in autophagy, apoptosis, and metabolism [2,14,15]. 

In our study, ELF-MF exposure at 50 Hz, 1 mT (24 h on/48 h off) did not affect the PBMC proliferation rate, cell cycle progression, or the activation of apoptotic pathways. The impact of ELF-MF on cell proliferation, viability, and apoptosis appears to vary depending on the cell type and exposure parameters [16,17]. There is growing evidence that cellular responses are influenced not only by magnetic flux density, but also by the overall characteristics of ELF-MF exposure, including frequency, intensity, duration, and whether the exposure is continuous or intermittent [10,15,16,18,19,20,21]. These factors may help explain the variability in previous findings regarding PBMC proliferation, with studies reporting no effect, inhibitory effects, or stimulatory effects. For example, no significant changes in lymphocyte activation and proliferation were observed after exposure to 50 Hz sinusoidal magnetic fields at 0.05 mT and 2.5 mT [22]. Similarly, exposure up to 500 µT did not affect PBMC cytotoxic activity or cytokine production [23]. However, reduced NK cell activity has been reported in individuals occupationally exposed to ELF-MF, suggesting possible long-term effects [24]. At higher magnetic flux densities, 50 Hz ELF-MF has been shown to inhibit proliferation and viability in human lymphoma cells (≥2.5 mT), as well as lymphocyte viability (35 mT) [25,26]. Conversely, some studies reported a slight increase in phytohemagglutinin-induced lymphocyte proliferation, indicating that ELF-MF may exert synergistic effects with mitogen stimulation, becoming evident only in activated lymphocytes [27,28]. In addition, radiofrequency (RF) fields amplitude-modulated at 60 Hz have been shown to transiently suppress T cell cytotoxicity during the early phases of immune recognition [29]. Since modulated RF signals frequently coexist with ELF-MF near power lines, the potential combined effects of 50/60 Hz ELF-MF and RF fields should be considered when evaluating the impact of ELF-MF exposure from electric network on immune function [30]. 

In our study, a comparative proteomic analysis between ELF-MF–exposed and unexposed PBMCs revealed that ELF-MF exposure significantly altered the proteomic profile of the cells. Specifically, ELF-MF exposure led to the upregulation of 135, 179, and 339 proteins in samples from the three analyzed subjects, respectively. Among the upregulated proteins, SLC25A4, a mitochondrial inner membrane transporter essential for ATP synthesis, was consistently upregulated in all PBMC samples following ELF-MF exposure. This ADP/ATP antiporter facilitates the import of ADP into the mitochondrial matrix and the export of ATP, the primary energy currency of the cell [31]. Notably, SLC25A4 has also been reported to be upregulated in human PBMCs treated with vitamin D_3_, a key modulator of the immune system [32], and downregulated in PBMCs from patients with rheumatoid arthritis [33]. Of the 51 proteins found to be commonly upregulated in at least two out of the three subjects, 36 were associated with biological processes such as *Cellular Metabolic Process*, *Metabolic Process*, and *Organic Substance Metabolic Process*, according to Gene Ontology classification. These proteins were also involved in KEGG pathways related to *Starch and Sucrose Metabolism*, *Carbon Metabolism*, and the *Biosynthesis of Amino Acids*. STRING network analysis further indicated that these upregulated proteins participate in key Reactome pathways, including *Infectious Disease*, *Disease-Related Mechanisms*, and *HIV Infection*. 

An analysis of the 35 downregulated proteins revealed enrichment in Gene Ontology biological processes such as *T Cell Costimulation* and *Cell Activation*, both of which are critical for an effective immune response [34]. Proteomic data further showed that PCYT1A and PCYT2 were consistently downregulated in PBMC samples from all three subjects following ELF-MF exposure. PCYT1A and PCYT2 are key metabolic genes involved in phospholipid biosynthesis, indirectly shaping immune cell function by maintaining membrane integrity required for signaling, regulating energy metabolism in PBMCs, and modulating inflammatory responses [35,36,37]. Specifically, PCYT1A encodes an essential enzyme for the synthesis of phosphatidylcholine (PC), a phospholipid vital for membrane structure and immune cell signaling. Disruption in PC synthesis in macrophages has been shown to alter membrane lipid composition, regulate toll-like receptor 4 (TLR4) signaling, and affect pro-inflammatory cytokine secretion [37,38,39,40]. ELF-MF also downregulates PCYT2, which plays a crucial role in phosphatidylethanolamine (PE) synthesis, essential for membrane integrity and function [41]. PE regulates macrophage functionality and inflammatory responses [42,43] It is enriched in mitochondrial membranes, and its disruption can impair T-cell energy metabolism and antiviral responses [44,45]. T cells deficient in PCYT2 exhibit defective phospholipid metabolism and reduced humoral immune responses [46]. In addition to PCYT1A and PCYT2, three other proteins, ASPSCR1, QARS1, and REPS1, were downregulated in PBMCs from all three subjects. ASPSCR1 is involved in intracellular trafficking of GLUT4, thereby impacting glucose uptake and overall cellular metabolism, which in turn may modulate immune cell function [47,48]. QARS1 is a cytoplasmic enzyme essential for protein synthesis, catalyzing the attachment of glutamine to its corresponding tRNA. Mutations in QARS1 have been linked not only to neurological disorders but also to impaired lymphocyte proliferation [49,50]. Additionally, QARS1 has been shown to interact with the HIV-1 Gag protein, suggesting a possible role in viral processes [51]. REPS1 has been implicated in the regulation of membrane protein trafficking, a process potentially influencing T cell receptor endocytosis, MHC molecule recycling, and PD-1 surface expression in exhausted T cells [52,53]. 

Although the IARC classified ELF-MF in Group 2B (“possibly carcinogenic to humans”) in 2002 [54] subsequent assessments by the World Health Organization (WHO) and guidelines issued by the International Commission on Non-Ionizing Radiation Protection (ICNIRP) have concluded that public exposure levels below 100 μT can be considered safe [55,56,57]. However, emerging research has raised concerns regarding the adequacy of current occupational exposure limits (typically 0.5–1 mT) for workers who are regularly exposed to higher ELF-MF magnetic flux densities at 50/60 Hz. Several studies have reported measurable biological changes at these elevated levels, suggesting the potential for long-term health risks and challenging the assumption that current exposure guidelines are universally protective. The immune system appears to be particularly sensitive to sustained ELF-MF exposure. Multiple studies have demonstrated significant reductions in NK cell activity and the modulation of innate immune cytokine expression [58,59]. Metabolic disturbances are equally concerning. A growing body of evidence indicates that sustained high-flux-density ELF-MF exposure may disrupt cellular energy production, with studies identifying cellular metabolism as one of the primary targets of the cellular biological response to ELF-MF exposure. ELF-MF can induce mitochondrial dysfunction and impact energy metabolism. [2,10,60]. ELF-MF exposure has been shown to affect the expression of genes involved in glucose and fatty acid metabolism, as well as to alter serum lipid profiles [61,62]. However, the mechanisms underlying these alterations remain only partially understood. From the standpoint of classical physics, such biological effects of ELF-MF appear difficult to explain [63]. This has prompted the exploration of alternative frameworks, including quantum physics, to better account for these interactions in biological systems, such as those involving zwitterions [64,65,66]. 

The flux density of 1 mT selected for this study corresponds to the low action level established by the European Directive 2013/35/EU for occupational exposure to 50 Hz ELF-MF [67,68]. This threshold represents the minimal level at which preventive measures are recommended in workplace settings, making it a critical benchmark for evaluating potential biological effects relevant to worker safety. While the directive permits higher exposure levels (up to 6 mT), these are less common and typically occur only in specific industrial environments. By focusing on the low action threshold, our study aims to provide insight into the biological impact at the earliest point of regulatory concern, thereby supporting risk assessment and occupational health policies. It should be noted that some of the observed effects at 1 mT may be relevant to populations exposed to lower flux densities, such as the general public, for whom exposure limits are typically set around 100 µT. Although the exposure levels differ by an order of magnitude, similar biological mechanisms could be involved, suggesting that findings at 1 mT might provide useful information for understanding potential effects at lower exposure levels.

This study has several limitations that should be considered when interpreting the findings. First, the sample size was limited to three individuals. Although a paired design was employed to minimize interindividual variability, the small cohort size limits the generalizability of the results. Nevertheless, the analysis focused on detecting robust and consistent molecular changes across individuals, which may reduce the risk of false-positive associations. Second, the ex vivo treatment model does not fully recapitulate the complexity of in vivo systemic physiological responses. As such, while the model is suitable for identifying direct biochemical alterations at the protein level, it may not capture downstream effects or broader network-level interactions that occur in vivo. Third, only a single exposure duration and one flux density of ELF-MF were investigated. This limits the generalizability and interpretability of the findings, as different exposure times or flux densities may induce distinct biological effects. These limitations highlight the need for studies in larger cohorts and relevant in vivo models, as well as for investigating a wider range of exposure conditions to clarify a possible dose–response relationship. This study highlights that, while exposure to 50 Hz, 1 mT ELF-MF does not significantly alter PBMC proliferation or viability, it induces distinct proteomic modifications. Bioinformatic pathway analysis reveals an upregulation of core metabolic processes, coupled with a downregulation of immune-related pathways. The upregulation of SLC25A4, a mitochondrial ADP/ATP translocase, suggests the activation of compensatory mechanisms to maintain bioenergetic homeostasis following ELF-MF-induced mitochondrial stress. 

The concurrent downregulation of PCYT1A and PCYT2, which encode rate-limiting enzymes in phospholipid biosynthesis, indicates the potential disruption of membrane-dependent immune signaling pathways. These molecular adaptations, while not cytotoxic, may have long-term implications for immune cell function, particularly in scenarios requiring robust immune activation.

Although these findings demonstrate ELF-MF’s capacity to influence cellular physiology at subtoxic levels, further research is necessary to determine whether such modifications could affect immune competence over prolonged exposures, particularly in vulnerable populations.

## 4. Materials and Methods

### 4.1. Donors and Blood Samples

All participants were male blood donors affiliated with the transfusion center of the National Health Service (NHS) hospital wards in Ancona (Italy), undergoing their routine blood donation. Enrollment criteria included healthy adults aged 25 to 45 years who had abstained from the use of any synthetic drugs, natural remedies, or dietary supplements (including vitamins and minerals) as well as from receiving any vaccinations within the 30 days prior to enrollment. Participants were required to be free from acute or chronic degenerative diseases, including cardiovascular conditions, metabolic disorders (e.g., hypercholesterolemia, diabetes, hyperuricemia), chronic viral infections, malignancies, autoimmune diseases, and psychiatric disorders. A limited number of subjects (*n* = 3) was deemed acceptable for the identification of large and consistent proteomic changes induced by ELF-MF under ex vivo conditions. A paired pre- and post-treatment design was used to minimize interindividual variability and enhance the detection of consistent protein-level changes. All participants followed a varied, unrestricted diet, and no familial relationships were present among them. Participant characteristics are detailed in Table 1. All participants provided written informed consent prior to inclusion in the study. All procedures were approved by the local ethics committee (Prot. no. 210340, 8/7/2010) and followed the Declaration of Helsinki guidelines.

### 4.2. PBMC Isolation and Culture

A 10 mL sample of venous whole blood was collected from each subject enrolled in the study using EDTA-coated tubes (Becton Dickinson, Wokingham, UK). PBMCs were isolated by density gradient centrifugation using a Ficoll–Biocoll separation solution (MP Biomedicals, Santa Ana, CA, USA), according to the manufacturer’s instructions. Briefly, whole blood was diluted with PBS (Biowest, Nuaillé, France) in a Falcon tube (Euroclone, Milan, Italy) and centrifuged at 1500 rpm for 30 min at room temperature without break. The PBMC-containing interphase was carefully aspirated using a graduated pipette and transferred to a new centrifuge tube. Isolated PBMCs were washed with PBS and centrifuged at 2000 rpm for 10 min at room temperature. The cell pellet was resuspended in RPMI-1640 medium supplemented with 10% heat-inactivated fetal bovine serum (FBS; Euroclone), 2 mM L-glutamine, and 100 U/mL penicillin-streptomycin (Euroclone).

### 4.3. PBMC Exposure to ELF-MF

The characteristics of the Helmholtz coil system used to generate the 50 Hz, 1 mT ELF-MF were previously described [2]. The apparatus was specifically engineered to expose cells within a standard incubator. To account for the thermal insulation properties of the incubator, the coil was designed to minimize heat generation. The copper wire gauge was selected to ensure that power dissipation did not exceed 8 Watts, thereby preserving the incubator’s ability to maintain a stable temperature. The coil dimensions (inner side length: 34 × 34 cm) and the distance between the two solenoids (19.5 cm) were determined based on simulations and tests previously reported in the literature [69]. The ELF-MF generated by the system was validated using a professional magnetic field analyzer (EFA-300; Wandel & Goltermann, Germany). PBMCs isolated from each volunteer were immediately divided into control samples (unexposed PBMCs) and ELF-MF-exposed samples (exposed PBMCs). Cells were seeded in 6-well plates at a density of 1 × 10^6^ cells/well. ELF-MF-exposed PBMCs were placed immediately after isolation at the center of the Helmholtz coil, which was situated inside a temperature- and atmosphere-controlled incubator (37.0  ±  0.1 °C, 5% CO_2_). Control samples were maintained in a separate incubator without ELF-MF exposure (the background 50 Hz ELF-MF generated by the incubator was 0.792 μT). To exclude potential thermal effects, the temperature at the level of the cell cultures was continuously monitored in both experimental conditions using a Thermochron iButton DS1922L (Maxim Integrated, San Jose, CA, USA). The temperature difference between the exposed and control samples was consistently maintained below 0.1 °C throughout the experiment. Following 24 h of ELF-MF exposure, the treated PBMCs were promptly transferred to the same incubator as the control group, where both groups were maintained for an additional 48 h prior to analysis (cell counting, flow cytometry, and protein extraction for proteomics). The 48 h post-exposure interval allows time for the activation and propagation of signaling pathways, transcriptional responses, and post-translational modifications that can result in measurable proteomic changes. Furthermore, delayed assessment may help distinguish stable, biologically meaningful alterations from transient, non-specific stress responses.

### 4.4. Cell Counting

Cell counts were performed using a Bürker chamber under a light microscope (Zeiss, Milan, Italy). A total of 10 µL of each cell suspension, after gentle mixing, was loaded into the chamber, and cells within the defined grid area were counted.

### 4.5. Flow Cytometry

Flow cytometry was performed using a FACSCanto II flow cytometer (BD Biosciences, Franklin Lakes, NJ, USA) equipped with BD FACSDiva software version 9.0. Viability and apoptosis were assessed using the Annexin V-FITC Apoptosis Detection Kit (eBioscience™, Thermo Fisher Scientific, Inc., Milan, Italy), following the manufacturer’s instructions. Cells were washed with PBS (Biowest, Nuaillé, France) and incubated for 15 min at room temperature in the dark with fluorochrome-conjugated Annexin V in 1× Binding Buffer. After a subsequent wash in 1× Binding Buffer, cells were incubated for 15 min with a Propidium Iodide (PI) staining solution. Samples were analyzed immediately after incubation, without additional washing.

For cell cycle analysis, 1 × 10^6^ cells were fixed in cold (2–8 °C) 70% ethanol overnight. After fixation, cells were washed twice with cold PBS and centrifuged at 2000 rpm for 10 min; the supernatant was discarded. Cells were then incubated at room temperature for 30 min with PI solution (Miltenyi Biotec, Bergisch Gladbach, Germany) at a final concentration of 1 µg/mL, supplemented with 1 U of RNase (DNase-free). Samples were analyzed immediately after incubation without further washing.

### 4.6. Protein Extraction, Digestion, and Peptide Preparation for Mass Spectrometry-Based Proteomics

Unexposed and ELF-MF-exposed PBMCs were washed with 1 × PBS and incubated in Urea buffer 8 M-Tris 100 mM HCL 30 min on ice and mix by vortex every 10 min. Lysates were centrifuged at 16,000× *g* for 20 min (Eppendorf^®^ Microcentrifuge 5415, Eppendorf, Hamburg, Germany; Merck KGa) at 4 °C. Protein concentration measurement were performed using a Bradford assay (Merck KGaA, Darmstadt, Germany). The sample protein (25 μg) was resuspended in 8M Urea 10 mM Tris-HCl buffer and was reduced by TCEP and alkylated using chloroacetamide [70]. Proteins were digested using Lys-C and trypsin, desalted in a C18 Stage Tip (Thermo Fisher Scientific) and peptides were resuspended in 20 μL Solvent A (2% acetonitrile, 0.1% Formic Acid).

### 4.7. Mass Spectrometry Analysis

Label-free quantification (LFQ) enables extensive proteome coverage and broad dynamic range for large-scale proteomic studies without the need for stable isotope labeling. This approach is recognized as robust and reliable for the relative quantification of proteins in complex biological matrices [71].Mass spectrometry analysis was performed using a quadrupole Orbitrap Q-Exactive HF mass spectrometer (Thermo Fisher Scientific) equipped with an LC–ESI–MS/MS interface. For each sample, 5 µL was analyzed in technical replicates. Peptides were separated using a linear gradient from 95% Solvent A (2% acetonitrile, 0.1% formic acid) to 30% Solvent B (80% acetonitrile, 0.1% formic acid) over 210 min, followed by an increase to 60% Solvent B in 20 min and a final ramp to 100% in 2 min. The flow rate was maintained at 0.25 µL/min using a UHPLC Easy-nLC 1000 system (Thermo Fisher Scientific) coupled to a 23-cm fused-silica emitter (75 µm inner diameter, New Objective, Inc., Woburn, MA, USA). The emitter was packed in-house with ReproSil-Pur C18-AQ 1.9 µm beads (Dr. Maisch GmbH, Ammerbuch, Germany) using a high-pressure bomb loader (Proxeon, Odense, Denmark) as described by [72]. For MS spectra acquisition (300–1650 *m*/*z*), the resolution was set at 60,000 with an AGC target of 3 × 10^6^ and an injection time of 20 ms. For HCD MS/MS spectra, the resolution was 15,000 at *m*/*z* 200, with an AGC target of 1e5, injection time of 80 ms, normalized collision energy (NCE) of 28%, and isolation width of 2.0 *m*/*z*. Raw data were processed using MaxQuant (version 1.5.2.8) [73]. Peptide identification was carried out via the Andromeda search engine against the UniProt Homo sapiens database (98,036 entries) [74]. Mass tolerances were set at 5 ppm for precursor ions and 20 ppm for fragment ions. Both peptide and protein false discovery rates (FDRs) were controlled at 1%, and only peptides with a minimum length of six amino acids were considered. Reverse hits and known contaminants were excluded. LFQ was performed using intensity values normalized across the dataset, with a minimum ratio count of 2 and the “match between runs” option enabled. 

Protein abundance levels in ELF-MF-exposed and control samples were analyzed using the Perseus platform (http://www.coxdocs.org; accessed on 16 September 2024) [75,76]. Missing values were imputed using values drawn from a normal distribution (width 0.3, downshift 1.8) on a per-column basis. LFQ quality control was conducted by assessing the consistency among replicates. Specifically, we employed the LFQ-Analyst tool [12] to: (i) calculate the Coefficient of Variation (CV), which quantifies the variability relative to the mean within our dataset (median CV < 20% indicates good reproducibility), and (ii) perform a Principal Component Analysis (PCA) to evaluate the similarity between technical replicates and to highlight differences between control and exposed samples. Hierarchical clustering was performed by Perseus tool using Euclidean distance, average linkage, and 300 clusters for both row and column dendrograms.

### 4.8. Bioinformatics Analyses

Each differentially expressed protein identified by the analysis was assigned to the respective human official NCBI Gene Symbol identifier. Protein–protein interaction networks and functional enrichment analyses were performed using the STRING database (https://string-db.org/; accessed on 8 January 2025) to investigate potential biological relationships and pathway involvements.

### 4.9. Statistics

A paired Student’s *t*-test was used to assess the differences between the PBMCs exposed to ELF-MF and the unexposed control group. The results are presented as the mean ± standard deviation (SD). Statistical analyses were carried out using SPSS software, version 19.0 (SPSS Inc., Chicago, IL, USA). A *p*-value of less than 0.05 was considered statistically significant. A further differential expression analysis between control and ELF-MF-treated groups was performed using the LFQ-Analyst tool. Volcano and box plots were generated using the same tool. Differentially expressed proteins were defined as those with |log_2_(fold change)| ≥ 1.5 between the exposed and unexposed control groups, with an adjusted *p*-value < 0.05 (Benjamini-Hochberg method for false discovery rate correction).

## Figures and Tables

**Table 1:** Demographic and clinical characteristics of the three PBMC donors.

	Subject #1	Subject #2	Subject #3
Age (years)	30	43	32
Sex	Male	Male	Male
Ethnic background	European	European	European
BMI (kg/m^2^)	23.9	23.5	26.3
Systolic blood pressure (mmHg)	130	110	120
Diastolic blood pressure (mmHg)	70	80	75
Fasting blood glucose (mg/dL)	88	92	85
Total cholesterol (mg/dL)	172	198	188
Triglycerides (mg/dL)	134	142	117
Alcohol consumption (grams/week)	24	36	12
Leisure-time physical activity (min/week)	120	60	180
Smoker	No	No	No
Family history of obesity	No	No	No

## Data Availability

The raw proteomic data have been uploaded to the jPOST repository with the identifiers JPST003837 (jPOST) and PXD064718 (ProteomeXchange).

## Supplementary Materials

The supporting information can be downloaded at: https://www.mdpi.com/article/10.3390/ijms26136035/s1.
