# Supplementary material for: Proteomic Characterization of Human Peripheral Blood Mononuclear Cells Exposed to a 50 Hz Magnetic Field
Source: Int J Mol Sci. 2025 Jun 24;26(13):6035. doi: 10.3390/ijms26136035 (PMC12250113; doi:10.3390/ijms26136035)
Supplement: Supplementary file 1 [file ijms-26-06035-s001.zip › ijms-3658684-supplementary.pdf]

SUPPLEMENTARY MATERIAL

"Proteomic Characterization of Human Peripheral Blood Mononuclear Cells Exposed to a 50 Hz Magnetic Field"

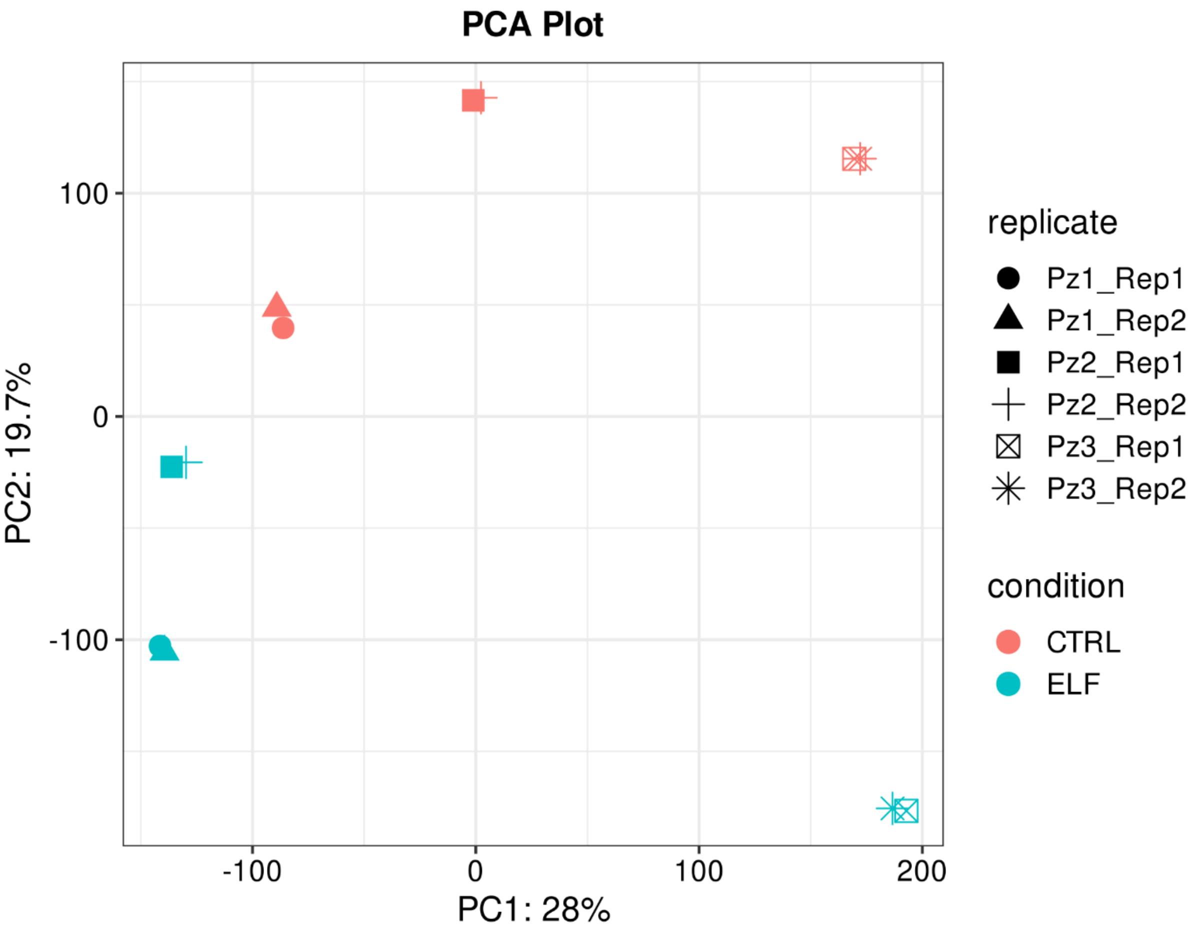

Figure S1. Principal Component Analysis (PCA) for LFQ data quality control of 12 samples derived from three patients, including six controls and six ELF-MF-exposed samples, each with two technical replicates. The tight clustering of technical replicates demonstrates high reproducibility, while the clear separation between control and exposed samples highlights the distinct effects of ELF-MF treatment.

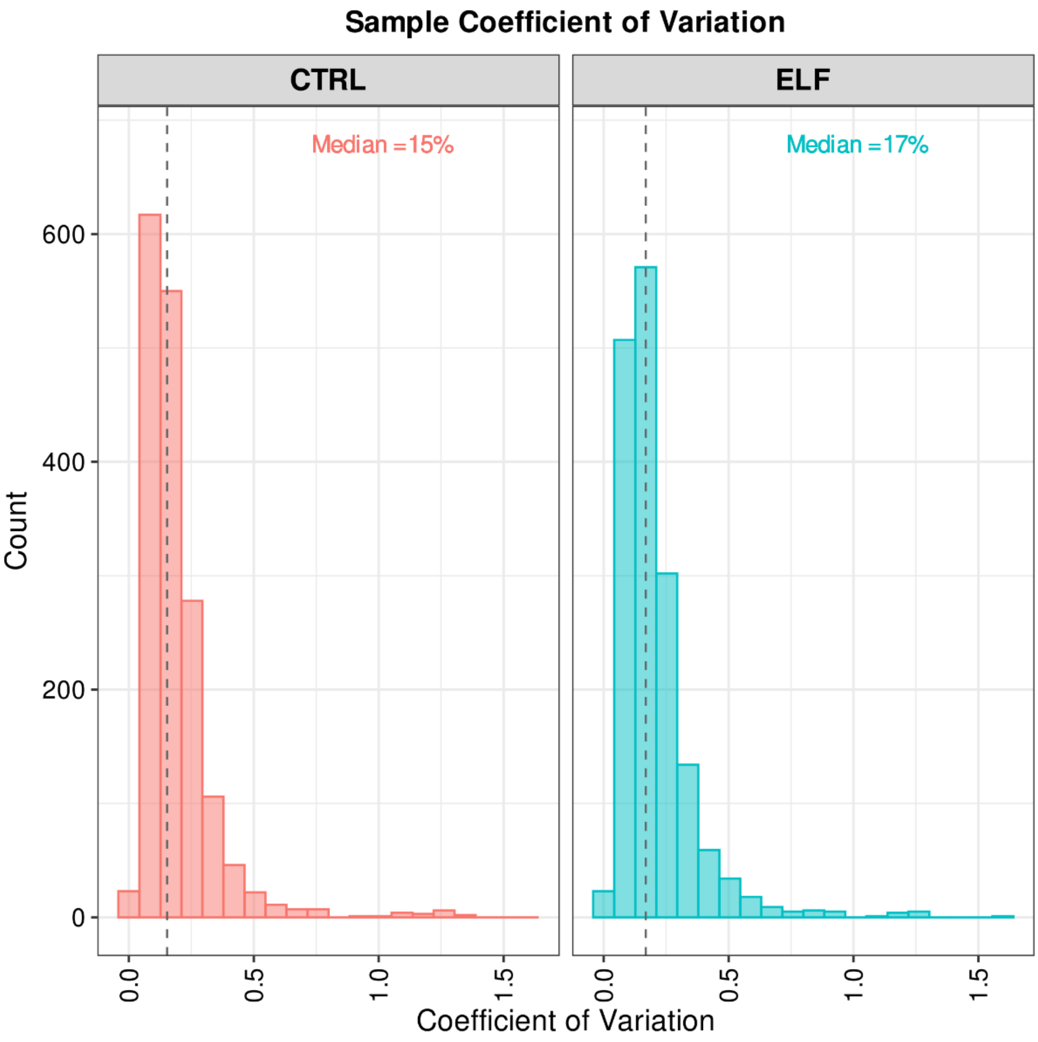

Figure S2. Distribution of coefficient of variation (CV) values in the control and ELF-MF-exposed groups, shown for LFQ data quality control. A median CV below 20% is widely accepted as indicative of high data reproducibility.

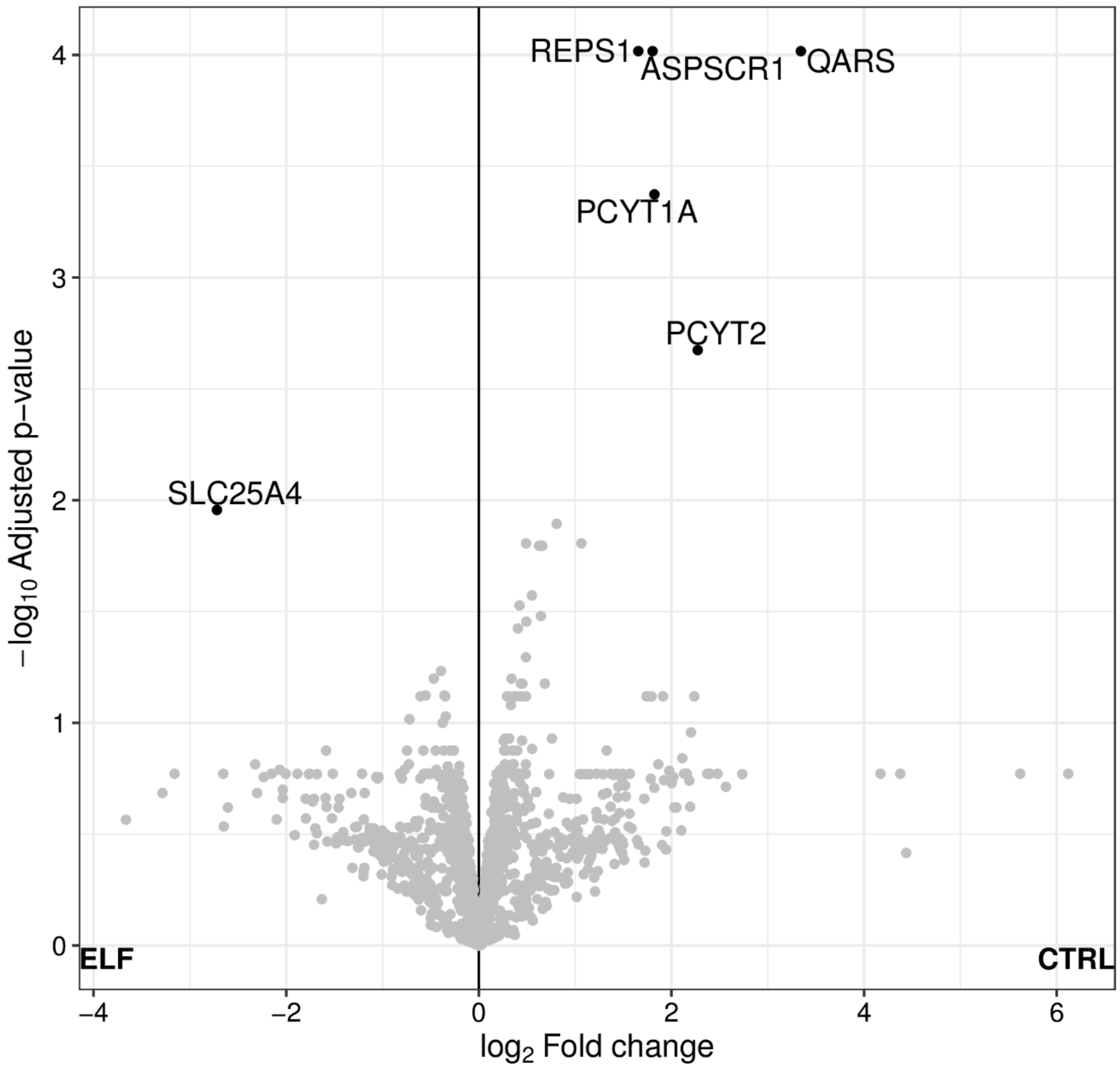

Figure S3. Volcano plot showing the fold changes and adjusted p-values for all quantified proteins, comparing ELF-MF-exposed samples to controls. Five proteins are significantly differentially expressed: SLC25A4 is upregulated, while ASPCR1, PCYT1A, PCYT2, QARS, and REPS1 are downregulated in treated samples. Proteins were considered differentially expressed if they had adjusted p-values < 0.05 and $|\log_2(\text{fold change})| \geq 1.5$.

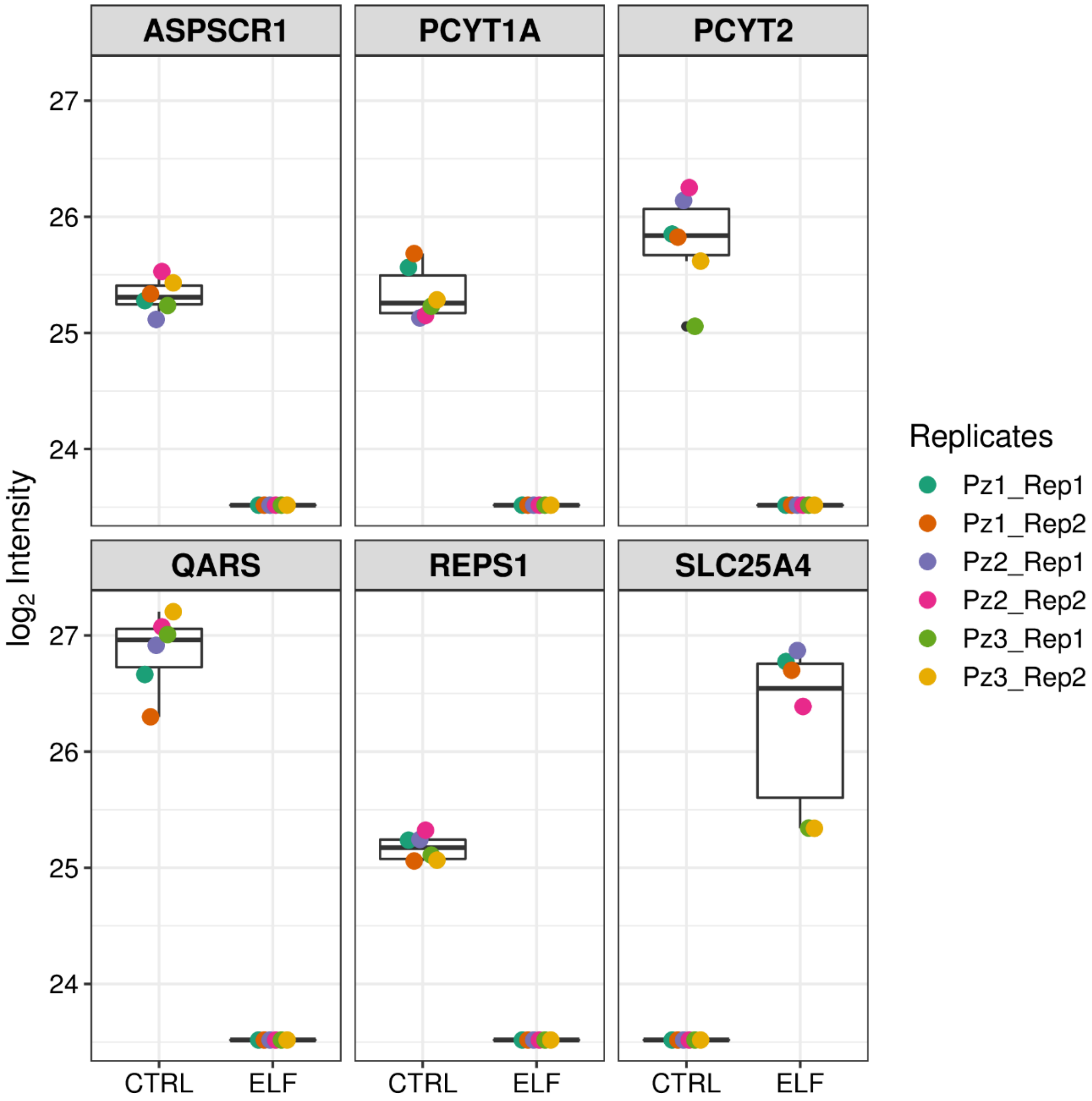

Figure S4. Box plots of the six differentially expressed proteins between ELF-MF-exposed and control samples. The plots highlight strong consistency across both biological and technical replicates and demonstrate a clear treatment-dependent effect on protein expression levels.

Table S1. The table provides an overview of the main characteristics of the differentially expressed proteins revealed by our analysis.

Gene Name	Protein ID	ELF-MF/CTRL log_2 fold change	p-value	Adjusted p-value	Protein names
REPS1	Q96D71	-1.66	1.26E-07	0.0000961	RalBP1-associated Eps domain-containing protein 1
QARS	Q53HS0	-3.34	1.45E-07	0.0000961	Glutamine--tRNA ligase
ASPSCR1	J3KRY8	-1.80	1.71E-07	0.0000961	Tether containing UBX domain for GLUT4
PCYT1A	C9J050	-1.82	0.000001	0.000423	Choline-phosphate cytidylyltransferase A
PCYT2	I3L1R7	-2.27	0.00000629	0.00212	Ethanolamine-phosphate cytidylyltransferase
SLC25A4	Q6I9V5	2.72	0.0000394	0.0111	Solute carrier family 25 member 4; ADP/ATP translocase 3; ADP/ATP translocase 3, N-terminally processed; ADP/ATP translocase 1
